# Awareness of Rheumatic Heart Disease in Egypt: A National Multicenter Study

**DOI:** 10.3390/jcdd8090108

**Published:** 2021-09-04

**Authors:** Ahmed Kamal Sayed, Hussien Se’eda, Nael Kamel Eltewacy, Loalo’a El Sherif, Hagar Samy Ghalioub, Ahmed Sayed, Ahmed M. Afifi, Hesham S. Almoallim, Sulaiman Ali Alharbi, Abdelrahman I. Abushouk

**Affiliations:** 1 Faculty of Medicine, Minia University, Minia 61782, Egypt; a.Kamal@s-mu.edu.eg (A.K.S.); hussien.mohamed231@s-mu.edu.eg (H.S.); loaloa.mohamad@s-mu.edu.eg (L.E.S.); hagersamy16@gmail.com (H.S.G.); 2 Minia Medical Research Society (MMRS), Minia University, Minia 61782, Egypt; nael.eltwacy@gmail.com; 3 Faculty of Pharmacy, Beni-Suef University, Beni-Suef 62514, Egypt; 4 Faculty of Medicine, Ain Shams University, Cairo 11591, Egypt; ahmedsayed8891@gmail.com; 5 Margret M. and Albert B. Alkek Department of Medicine, Baylor College of Medicine, Houston, TX 77030, USA; drafifi1993@gmail.com; 6 Department of Oral and Maxillofacial Surgery, College of Dentistry, King Saud University, P.O. Box 60169, Riyadh 11545, Saudi Arabia; hkhalil@ksu.edu.sa; 7 Department of Botany and Microbiology, College of Science, King Saud University, P.O. Box 2455, Riyadh 11451, Saudi Arabia; sharbi@ksu.edu.sa; 8 Department of Cardiovascular Medicine, Heart, Vascular and Thoracic Institute, Cleveland Clinic, Cleveland, OH 44195, USA

**Keywords:** rheumatic fever, rheumatic heart disease, knowledge, survey, Egypt

## Abstract

Background: While the prevalence of rheumatic heart disease (RHD) in Egypt is relatively high, data on the awareness of Egyptians about the cause of RHD are lacking. Methods: Using a pre-tested questionnaire, we performed a multicenter survey of outpatients attending 15 university hospitals across Egypt. Results: A total of 6958 participants were interviewed. Most subjects (81.7%) reported a previous experience of sore throat. Seeking treatment, most patients (69.3%) consulted a medical professional, while the others relied on self/peer medication. Individuals consulting a physician received antibiotics more frequently than those who did not (89.1 vs. 38.7%; OR: 12.4, 95% CI 10.8–14.1). The median RHD knowledge score in our sample was 4 (IQR = 6). While most subjects (56.3%) claimed knowledge of the complications of an untreated sore throat, only a third (34%) were aware of the association between sore throat and RHD. In a multivariate analysis, older age (Mean Difference [MD]: 1.58, 95% CI 1.37–1.79), female gender (MD: 0.89, 95% CI 0.75–1.04), higher education (MD: 1.10, 95% CI 0.90–1.30), and being interviewed outside Cairo (MD: 0.67, 95% CI 0.51–0.82) were significant predictors of knowledge about RHD. Conclusion: The current study showed low levels of awareness on the cause of RHD among Egyptians and highlights a pressing need for interventions to address this public knowledge gap.

## 1. Introduction

Rheumatic heart disease (RHD) is a long-term cardiac inflammatory disorder that results from a single severe or multiple recurrent attacks of acute rheumatic fever (ARF). This is an abnormal immune response that follows an improperly managed group A streptococcus (GAS) infection in a genetically susceptible host [1]. Pharyngitis is the most common presentation of GAS infection in children between 5 and 15 years old; however, GAS is identified as the causative pathogen in only 15 to 20% of pharyngitis cases. Nearly 60% of ARF patients in endemic areas develop RHD. The prevalence of RHD is highest in adults aged 25 to 45 years, which reflects late detection and the long-term effects of ARF attacks [2].

About 34.2 million people live with RHD worldwide, resulting in 345,110 deaths and 10.1 million disability-adjusted life years lost annually. These numbers are probably underestimated due to limited global data, underdiagnosis, and the lack of formal reporting systems [3,4,5]. Although the incidence rates of ARF and RHD have decreased globally (specifically in developed countries), it remains a major health burden in low and middle-income countries, including Egypt [6]. According to the Egyptian Ministry of Health’s 2018 report, Egypt has around 300,000 RHD patients aged between 5 and 15 years [7,8]. According to the World Health Organization’s 2014 statistics, Egypt had the highest total deaths (*n* = 9168) among the East Mediterranean countries [9]. An echo-based screening study showed that the prevalence of RHD is 31 cases per 1000 school children in Aswan, Egypt [10], which is higher than that found in Cape Town, South Africa (20 per 1000 [11]) and Kampala, Uganda (15 per 1000 [12]).

RHD is a poverty-stricken disease with a higher burden in communities with high illiteracy rates and poor access to healthcare resources. According to Egypt’s Central Agency for Public Mobilization and Statistics, the poverty rate in Egypt during 2019/2020 was 29.7%, with higher concentrations in rural areas. According to the World Bank and UNESCO, the illiteracy rate in Egypt in 2017 was 28.8%, with higher rates in females and citizens aged 65 years and older. In terms of access to healthcare, the 2019 Global Burden of Disease study estimated that Egypt has a score of 55 on the universal health coverage index (a scale of 0 to 100) [13]. Multiple efforts are underway to expand universal health coverage in the country. Primordial prevention through improving living conditions, personal hygiene, and access to clean water, as well as increasing awareness and education has an important role in controlling and eradicating RHD [14]. Increased awareness allows for an earlier diagnosis and the adequate treatment of GAS infections, which are essential to prevent RHD [11,15]. In the present study, we aimed to assess the level of awareness about the cause of RHD in individuals attending the outpatient departments of different university hospitals across Egypt.

## 2. Materials and Methods

### 2.1. Study Design

The present study is a multicenter, cross-sectional survey that was conducted in the outpatient departments of 15 university hospitals across Egypt (Ain Shams University, Al-Azhar University for boys in Cairo, Assiut University, Aswan University, Beni-Suef University, Cairo University, Fayoum University, Menoufia University, Mansoura University, Kafrelsheikh University, Minia University, Misr University for Science and Technology (MUST), October 6 University, Sohag University, Tanta University). The study was conducted during the first two weeks of August 2019.

### 2.2. Study Participants

We included all potential participants (adults and older children of both sexes (>9 years old), attending the outpatient department for any reason. Verbal informed consent was taken from all respondents; for those <18 years of age, guardian consent was collected. The participants were briefly informed about the objective and benefits of participating in the study. The respondents did not essentially have clinical or subclinical RHD, nor a past history of ARF or a family history of RHD. We excluded people who were critically ill, people who came in with emergency medical conditions, and those who refused to participate.

### 2.3. Data Collection Tool

We used a structured questionnaire to evaluate the participants’ awareness about the different aspects of ARF and RHD. The questionnaire was obtained from a recently published study conducted in Cameroon [16] after obtaining approval from the authors. The questionnaire consisted of two parts: the first part collected demographic information, and the second part evaluated the participants’ knowledge on the association between sore throat infections and RHD. The questions in the second part assessed the participants exposure to sore throat attacks, medication practices and sources of healthcare advice, knowledge on the possible complications of sore throat/cause of RHD, and the preventive measures of RHD.

The questionnaire was translated from English to Arabic by two native Egyptian speakers and validated by a third Egyptian individual. To ensure accuracy, a back translation of the Arabic version to English was conducted. The questionnaire was pre-tested on 20 random participants to confirm the practicability, validity, and the interpretation of the responses. Most of the questions were simple ’yes’, ’no’, or ‘I don’t know’ answers. The survey was administered via face-to-face interviews by medical students with a good knowledge of ARF and RHD after group training to ensure conformity in question formatting and delivery. It took less than 10 min to be completed by each participant. There were one to three data collectors in each hospital. The subjects were only contacted at the point of conducting the surveys; no further contact was needed. 

### 2.4. Statistical Analysis

We summarized the continuous variables as median (interquartile ranges) or mean ± standard deviations. Overall knowledge was a variable constructed based on five questions, with each correct answer assigned a score of 2 and incorrect/”do not know” responses assigned a score of 0 (maximum score of 10). For continuous outcomes, we used an ordinary-least squares (OLS) regression to calculate the mean differences using both univariate and multivariate models. For binary outcomes, we used a logistic regression model to calculate the odds ratio using both univariate and multivariate models. Continuous variables were categorized according to quartiles if assumptions of linearity were not met. Significance tests were two-sided, and a *p*-value of 0.05 was considered statistically significant. Analyses were performed using R, version 4.0.3. We used the R packages “dplyr” [17] and “tidyr” [18] for data formulation and the “rms” package for constructing regression models [19].

### 2.5. Ethical Considerations

This study was approved by the institutional review board (IRB)—Faculty of Medicine, Minia University (IRB number: 224:7/2019) and conformed to the provisions of the Declaration of Helsinki in 1995. The survey was completely anonymous; responses were confidential and data from this research were reported only as a combined total.

## 3. Results

### 3.1. Demographics

We interviewed 6958 people with a median age of 33 (IQR: 22) years old. Only 32.1% of respondents were interviewed in Cairo. Most participants (63.5%) were females and the majority had either completed secondary (27.3%) or post-secondary (35.5%) education; however, approximately one fifth of the respondents were uneducated (defined as not having undergone at least primary education) (21.5%). Table 1 presents the detailed characteristics of our study participants.

### 3.2. Exposure

Most subjects (81.7%) reported a previous personal experience of at least a single sore throat attack and the majority (72.7 %) also reported that their children had experienced sore throat before. Further, more than half (57.2%) reported knowing people who had frequent sore throat attacks (>3 times a year). Younger age, female gender, and living in Cairo were associated (*p* < 0.001) with higher frequencies of reporting sore throat attacks.

### 3.3. Medication Use

To manage the infection, most patients (69.3%) consulted a doctor or a medical professional. However, nearly a quarter (25.8%) relied on self-medication, while a small minority (4.9%) received medications from a friend or a family member. Most participants (73.3%) reported using an antibiotic. Individuals that consulted a medical professional received antibiotics more frequently than those who did not (89.1 vs. 38.7%). This finding held true in both the univariate (OR: 12.9; 95% CI 11.4 to 14.7) and multivariate (OR: 12.4; 95% CI 10.8 to 14.1) analyses. In addition, individuals aged 23 to 45 were more likely to use an antibiotic, whereas those interviewed outside Cairo were less likely to report receiving one. In terms of physician consultation, younger people, males, and those interviewed in Cairo were less likely to have consulted a medical professional. Surprisingly, uneducated individuals in our sample were more likely to consult a medical professional than educated individuals. Detailed results of the univariate and multivariate logistic regression can be found in Table 2.

### 3.4. Knowledge on the Association between Sore Throat and RHD

We assessed the respondents’ knowledge on the association between RHD and sore throat. We found that although most respondents (62%) knew about RHD, only a small fraction (1.9%) correctly identified the cause (an open-ended question). Similarly, while the majority (56.3%) claimed to be aware of the complications of an untreated sore throat, only a third (34%) were aware of the association between sore throat attacks and heart disease. Yet, half (52.5%) of the respondents agreed that adequate sore throat treatment can prevent heart disease. Overall, our participants scored a median of 4 (IQR = 6) in terms of their overall knowledge of RHD.

Older individuals scored higher than younger ones, with the oldest age group scoring the highest (MD: 1.58, 95% CI 1.37 to 1.79). On average, females were significantly more knowledgeable than males (MD: 0.89, 95% CI 0.75 to 1.04), whereas people interviewed outside Cairo were significantly more knowledgeable than their counterparts (MD: 0.66, 95% CI 0.51 to 0.82). These associations were robust in both the univariate and multivariate models. For educational status, both secondary (MD: 0.58, 95% CI 0.37 to 0.78) and post-secondary education (MD: 1.10, 95 % CI 0.90 to 1.30) resulted in significant improvements in our multivariate regression, with the latter resulting in a greater increase. Detailed results of the univariate and multivariate linear regression can be found in Table 3.

## 4. Discussion

To our knowledge, this is the first large-scale study assessing knowledge on the association between sore throat and RHD in Egypt, a country with high prevalence rates of RHD [20]. In a cross-sectional survey of 6958 subjects, our findings revealed that a strikingly low proportion of participants correctly identified the cause of RHD, as well as a prevalent reliance on self/peer-prescribed medications. Interestingly, females were more likely to consult physicians and were more knowledgeable about the association between sore throat and RHD. Moreover, younger individuals (highest risk category) scored the lowest in terms of knowledge and were the least likely to consult a physician [21].

Our results on the poor public knowledge on the association between sore throat and RHD are similar to previous reports. Saeed and colleagues showed that among RHD patients in Pakistan, only 5% were aware that a sore throat was the cause of disease [22]. Another study by Nkoke et al. in Cameroon showed that only 5.1% of participants interviewed at outpatient clinics had adequate knowledge on RHD and that about 18.8% knew that sore throat infection precipitates RHD [16]. Similar results were reported in Tanzania [23]. In contrast, a study from New Zealand based on interviews with parents of children diagnosed with probable/possible RHD during a school-based screening showed that they had adequate knowledge and the majority knew that sore throat could cause rheumatic fever [24].

On the bright side, several studies have shown that health education can improve the awareness about the causes and management of RHD, both among the public and healthcare professionals. In an Indian study, 315 subjects were interviewed on the causes and consequences of sore throat and RHD before and after a health education program. The study showed that health education significantly improved the subjects’ awareness on the association between sore throat and RHD [25]. Another 10-year education program with central coordination and a multi-channel approach in two French Caribbean islands led to a >74% reduction in the incidence of ARF and a concurrent decrease in the incidence of glomerulonephritis (another post-streptococcal, immune-mediated condition) [26]. Among healthcare professionals, a Sudanese study showed that even doctors had an average knowledge on RHD, a finding that could be corrected with focused lectures [27]. Of course, raising public awareness alone is not sufficient and it should serve as a component of a multi-disciplinary effort, including evidence-based updates to the current guidelines and the utilization of more sensitive/specific tests in community screening for RHD.

The Pan-African Society of Cardiology devised the ASAP (Awareness, Surveillance, Advocacy, Prevention) program [11,28], which resulted in the REMEDY registry. This registry collected data from 3343 children in 12 African countries (including Egypt), India, and Yemen and highlighted gaps in secondary antibiotic prophylaxis, as well as in the management of those patients [29]. In Egypt, the national RHD prevention and control program, launched by the joint efforts of the WHO and the Egyptian Ministry of Health, analyzed the data of 17,050 subjects between 2006 and 2018 and showed that the incidence of RHD has fallen over time, probably due to improved living standards and access to healthcare resources in Egypt. However, the analysis showed a high rate of ARF/RHD misdiagnosis, requiring unaffected subjects to use long-term, injectable penicillin [8].

The main pillar of ARF/RHD prevention is the early antibiotic treatment of GAS infections. Therefore, access to primary healthcare services and consulting medical professionals is important. In our study, most patients (69.3%) consulted a doctor or a medical professional to manage their sore throat and the rate of antibiotic use was higher in patients consulting medical professionals than those who did not. This further underscores the role of physician utilization in the primary prevention of RHD. We should note, however, that while early antibiotic treatment of GAS infections can reduce the risk of ARF, antibiotics are not always the drug of choice for these patients, simply because GAS infections cause <20% of sore throat cases. Therefore, regulations should be enforced to prevent antibiotic misuse and the emergence of resistant strains, which is a rising threat in Egypt.

Another peculiar finding in our study was the inverse association between educational status and healthcare utilization. In a setting where unprescribed medications are widely available as in Egypt, individuals with higher education may feel more confident in their ability to self-medicate than less educated individuals. Nevertheless, studies have traditionally linked higher educational and socioeconomic status to increased healthcare utilization [30,31]. In addition, females were more likely to report utilizing physicians in our study, a finding consistent with previous reports [32,33,34].

In terms of knowledge about RHD, it was unsurprising to find that more educated individuals scored higher, though it is worth noting that this only applied to those completing secondary and post-secondary education. In addition, females and older individuals scored higher, which may be attributable to them being more likely to care for children and thus having greater awareness of pediatric health complications. This is especially likely in a more traditional society such as Egypt, where childcare falls squarely on the shoulders of mothers. In a former study by Vlajinac et al., low maternal educational status was an independent predictor of the incidence of rheumatic fever [35].

The limitations of our study include: (1) we could not collect data on other demographic variables, such as income, which may have been important to control for in our analysis; (2) despite the large sample size, our study may be less representative of the population than a community-based study. Future research should explore the determinants of knowledge about RHD and the inappropriate health-related behaviors in developing countries and investigate the efficacy of different public communication strategies for health messaging about RHD.

In conclusion, our study highlights a pressing need for strategies to increase awareness about RHD in Egypt, especially among the youth who are at a higher risk. Community-based campaigns that highlight the link between sore throats and RHD should be implemented with central coordination, collaborative design, and multi-channel messaging. Social media (Facebook, Instagram, Twitter, and YouTube) are valuable tools in health communication, especially with young adults. Finally, local communities should be actively engaged in the awareness process, especially in under-resourced locations. These efforts will help eradicate RHD in the 21st century.

## Figures and Tables

**Table 1:** Baseline Characteristics of the study participants.

Baseline Characteristic	
Total number of interviewees, *n*	6958 (100)
Age (Years), Median ± IQR	33 ± 22
Gender (Female), *n* (%)	4418 (63.5)
Educational Status, *n* (%)	
Uneducated	1494 (21.5)
Primary Education	832 (12.0)
Preparatory	259 (3.7)
Secondary	1900 (27.3)
Post-secondary	2473 (35.5)
Hospital Location, *n* (%)	
Cairo	2235 (32.1)
Other Governates	4723 (67.9)
Sore Throat Experience, *n* (%)	
Personal experience of a sore throat	5678 (81.7)
Child experienced sore throat	4794 (72.7)
Known a family member with frequent sore throat ^$^	3963 (57.2)
Reported receiving antibiotics for sore throat *	4767 (73.3)
Medications prescribed by a medical professional **	4487 (69.3)
Peer-prescriptions **	317 (4.9)
Self-prescriptions **	1670 (25.8)

^$^: percentage reported based on *n* of 6934, *: percentage reported based on *n* of 6500, **: percentage reported based on *n* of 6475.

**Table 2:** Association of physician consultation * with baseline characteristics.

		Univariate Model			Multivariate Model ^†^		
	*n* (% Consulting Physician)	OR	95% CI		OR	95% CI	
			LL	UL		LL	UL
Age (Years)							
Reference = First quartile (4 to 23)	1552 (59.7%)						
Second quartile (23 to 33)	1638 (69.4%)	**1.526**	**1.318**	**1.766**	**1.440**	**1.243**	**1.670**
Third quartile (33 to 45)	1592 (74.9%)	**2.016**	**1.731**	**2.347**	**1.851**	**1.583**	**2.164**
Fourth quartile (45 to 93)	1347 (69.7%)	**1.552**	**1.330**	**1.811**	**1.416**	**1.203**	**1.668**
Gender							
Reference = Male	2292 (66.4%)						
Female	4180 (70.9%)	**1.228**	**1.101**	**1.370**	**1.182**	**1.054**	**1.326**
Educational status							
Reference = uneducated	1330 (76.4%)						
Primary	770 (69.9%)	**0.717**	**0.587**	**0.875**	**0.792**	**0.642**	**0.977**
Preparatory	246 (60.2%)	**0.467**	**0.351**	**0.621**	**0.534**	**0.398**	**0.717**
Secondary	1747 (70.5%)	**0.737**	**0.626**	**0.868**	**0.812**	**0.682**	**0.967**
Post-secondary	2382 (65.2%)	**0.580**	**0.498**	**0.675**	**0.720**	**0.608**	**0.851**
Hospital Location							
Reference = Cairo	2099 (67.1%)						
Other Governates	4376 (70.4%)	**1.165**	**1.042**	**1.303**	**1.251**	**1.107**	**1.413**

*n* = Total Number of Interviewed individuals within each subgroup; % = Percentage of patients consulting a physician within each subgroup; OR = Odds Ratio; LL = Lower Limit; UL = Upper Limit; CI = Confidence Interval. Significant values are bolded. * Outcome data for appropriate physician consultation was available for 6475 respondents (missing = 483). ^†^ Adjusted for age, sex, educational status, and hospital location.

**Table 3:** Association of overall knowledge * of RHD with baseline characteristics.

	Univariate Model			Multivariate Model ^†^		
	MD	95% CI		MD	95% CI	
		LL	UL		LL	UL
Age (Years) ^††^						
Reference = First quartile (4 to 23)						
Second quartile (23 to 33)	**0.670**	**0.475**	**0.865**	**0.682**	**0.490**	**0.875**
Third quartile (33 to 45)	**1.084**	**0.887**	**1.281**	**1.273**	**1.076**	**1.471**
Fourth quartile (45 to 93)	**1.169**	**0.965**	**1.373**	**1.576**	**1.366**	**1.787**
Gender ^§^						
Reference = Male						
Female	**0.749**	**0.604**	**0.893**	**0.888**	**0.743**	**1.033**
Educational status						
Reference = uneducated						
Primary	**−0.308**	**−0.560**	**−0.057**	0.086	−0.169	0.341
Preparatory	−0.133	−0.524	0.257	0.107	−0.276	0.489
Secondary	0.180	−0.021	0.381	**0.577**	**0.369**	**0.785**
Post-secondary	**0.457**	**0.267**	**0.648**	**1.101**	**0.898**	**1.304**
Hospital Location						
Reference = Cairo						
Other Governates	**0.915**	**0.767**	**1.064**	**0.668**	**0.510**	**0.826**

RHD = Rheumatic Heart Disease; MD = Mean Difference; LL = Lower Limit; UL = Upper Limit; CI = Confidence Interval. Significant values are bolded. * Outcome data for overall knowledge of RHD was available for 6934 respondents (missing = 24). ^†^ Adjusted for age, sex, educational status, and hospital location. ^††^ Out of the 6934 respondents with available data, age was missing for 414 respondents. ^§^ Out of the 6934 respondents with available data, gender was missing for 2 respondents.

## Data Availability

Data are available from the corresponding author on reasonable request.

## Collaborators

Ain Shams University: Yasmin Hassan Elshoura, Heba Sayed Abdelazez Tesen, Kirollos Samuel Nassim Al-Seify; Al-Azhar University for boys in Cairo: Obada Atef Hassan, Asem Abdelfttah Soltan, Mohamed Elalem; Assiut University: Alaa Gamal Abd El Nasser, Esraa M. Hosny, Abdallah Tharwat Elmaghrabey, Ahmed Hamdi Awad; Aswan University: Ebrahim Farag, Anton Mohareb.; Beni-Suef University: Asmaa A. Zedan, Enas M. Yasseen; Cairo University: Ahmed O. Elmehrath, Shady Hany Fayek, Moaz Y. Farghal; Fayoum university: Alshaimaa Galal, Asmaa Khaled Mostafa, Reem Essam Sayed; Menoufia University: Ethar sabry, Salma Elnoamany; Mansoura University: Amany Eissa, Ahmed M. Shoman, Sara Lotf; Kafrelsheikh University: Mahmoud Safaan, Omar Marzook, Yasmeen El-Ezaby; Minia University: Mahmoud Ali Elshinawy, Sahar Ashraf Alnakib, Mariam Mahmoud Ahmad; Misr University for Science and Technology (MUST): Ingy Badawy, Mariam M. Eltelty, Mona A. Samy, Nada M. Abd El Fatah, Safa Elsayed; October 6 University: Khaled M. Hamam, Sarah Makram Elsayed, Youssof K. Elawdan; Sohag University: Ahmed Hassan Mahmoud, Mohamed El-Hammal, Mohamed Hashem Ahmed; Tanta University: Laila Salah Shamseldin, Sara Hamada Motawea, Hagar fathy Abdelrahman.

## Abbreviations

ARF	Acute rheumatic fever
GAS	Group A streptococci
RHD	Rheumatic heart disease

## References

[1] Carapetis J.R., Beaton A., Cunningham M.W., Guilherme L., Karthikeyan G., Mayosi B.M., Sable C., Steer A., Wilson N., Wyber R. (2016). Acute rheumatic fever and rheumatic heart disease. Nat. Rev. Dis. Primers.

[2] Zühlke L.J.L., Beaton A.A., Engel M.M., Hugo-Hamman C.C., Karthikeyan G., Katzenellenbogen J., Ntusi N.N., Ralph A.A., Saxena A.A., Smeesters P.R. (2017). Group A streptococcus, acute rheumatic fever and rheumatic heart disease: Epidemiology and clinical considerations. Curr. Treat. Options Cardiovasc. Med..

[3] Murray C.J.L., Barber R.M., Foreman K.J., Ozgoren A.A., Abd-Allah F., Abera S.F., Aboyans V., Abraham J.P., Abubakar I., Abu-Raddad L.J. (2015). Global, regional, and national disability-adjusted life years (DALYs) for 306 diseases and injuries and healthy life expectancy (HALE) for 188 countries, 1990–2013: Quantifying the epidemiological transition. Lancet.

[4] Marijon E., Mirabel M., Celermajer D.S., Jouven X. (2012). Rheumatic heart disease. Lancet.

[5] Zühlke L.J., Steer A.C. (2013). Estimates of the global burden of rheumatic heart disease. Glob. Heart.

[6] Vos T., Lim S.S., Abbafati C., Abbas K.M., Abbasi M., Abbasifard. M., Abbastabar H., Abd-Allah F., Abdelalim A., Abdollahi M. (2020). Global burden of 369 diseases and injuries in 204 countries and territories, 1990–2019: A systematic analysis for the Global Burden of Disease Study 2019. Lancet.

[7] Egyptian Ministry of Health and Population (2018). National RHD prevention and control Program. PLoS Negl. Trop. Dis..

[8] Ghamrawy A., Ibrahim N.N., Abd El-Wahab E.W. (2020). How accurate is the diagnosis of rheumatic fever in Egypt? Data from the national rheumatic heart disease prevention and control program (2006–2018). PLoS Negl. Trop. Dis..

[9] Abul-Fadl A.M.A.M., Mourad M.M., Ghamrawy A., Sarhan A.E. (2018). Trends in deaths from rheumatic heart disease in the eastern mediterranean region: Burden and challenges. J. Cardiovasc. Dev. Dis..

[10] Kotit S., Said K., ElFaramawy A., Mahmoud H., Phillips D.I.W., Yacoub M.H. (2017). Prevalence and prognostic value of echocardiographic screening for rheumatic heart disease. Open Heart.

[11] Engel M.E., Zuhlke L., Robertson K. (2009). Rheumatic fever and rheumatic heart disease: Where are we now in South Africa?: ASAP programme. SA Heart.

[12] Sanyahumbi A.S., Sable C.A., Beaton A., Chimalizeni Y., Guffey D., Hosseinipour M., Karlsten M., Kazembe P.N., Kennedy N., Minard C.G. (2016). School and community screening shows Malawi, Africa, to have a high prevalence of latent rheumatic heart disease. Congenit. Heart Dis..

[13] Lozano R., Fullman N., Mumford J.E., Knight M., Barthelemy C.M., Abbafati C. (2020). Measuring universal health coverage based on an index of effective coverage of health services in 204 countries and territories, 1990-2019: A systematic analysis for the global burden of disease study 2019. Lancet.

[14] Nordet P., Lopez R., Duenas A., Sarmiento L. (2008). Prevention and control of rheumatic fever and rheumatic heart disease: The Cuban experience (1986-1996-2002). Cardiovasc. J. Afr..

[15] Ramsey L.S., Watkins L., Engel M.E. (2013). Health education interventions to raise awareness of rheumatic fever: A systematic review protocol. Syst. Rev..

[16] Nkoke C., Luchuo E.B., Jingi A.M., Makoge C., Hamadou B., Dzudie A. (2018). Rheumatic heart disease awareness in the South West region of Cameroon: A hospital based survey in a Sub-Saharan African setting. PLoS ONE.

[17] Wickham H., François R., Lionel Henry K.M. (2020). dplyr: A Grammar of Data Manipulation R package version 1.0.2.

[18] Wickham H. (2020). tidyr: Tidy Messy Data. R package version 1.1.2.

[19] Harrell F.E. (2021). rms: Regression Modeling Strategies. R package version 6.2-0.

[20] Roth G.A., Mensah G.A., Johnson C.O., Addolorato G., Ammirati E., Baddour L.M., Baddour L.M., Barengo N.C., Beaton A.Z., Benjamin E.J. (2020). Global Burden of Cardiovascular Diseases and Risk Factors, 1990–2019: Update From the GBD 2019 Study. J. Am. Coll. Cardiol..

[21] Watkins D.A., Johnson C.O., Colquhoun S.M., Karthikeyan G., Beaton A., Bukhman G. (2017). Global, Regional, and National Burden of Rheumatic Heart Disease, 1990–2015. N. Engl. J. Med..

[22] Saeed M.H., Afzal M.S. (2016). Awareness of rheumatic heart disease in patients suffering from rheumatic heart disease. J. Univ. Med. Dent. Coll..

[23] Bergmark R., Bergmark B., Blander J., Fataki M., Janabi M. (2010). Burden of disease and barriers to the diagnosis and treatment of group a beta-hemolytic streptococcal pharyngitis for the prevention of rheumatic heart disease in Dar Es Salaam, Tanzania. Pediatr. Infect Dis. J..

[24] Gurney J.K., Chong A., Culliford-Semmens N., Tilton E., Wilson N.J., Sarfati D. (2017). High levels of rheumatic fever and sore throat awareness among a highrisk population screened for rheumatic heart disease. N. Z. Med. J..

[25] Arya R.K. (1992). Awareness about sore-throat, rheumatic fever and rheumatic heart disease in a rural community. Indian J. Public Health.

[26] Bach J., Chalons S., Mosser A., Forier E., Elana G., Jouanelle J., Kayemba S., Delbois D., Sainte-Aimé C., Berchel C. (1996). 10-year educational programme aimed at rheumatic fever in two French Caribbean islands. Lancet.

[27] Osman G.M., Abdelrahman S.M.K., Ali S.K.M. (2015). Evaluation of physicians’ knowledge about prevention of rheumatic fever and rheumatic heart disease before and after a teaching session. Sudan J. Paediatr..

[28] Mayosi B.M. (2010). The four pillars of rheumatic heart disease control. SAMJ S. Afr. Med. J..

[29] Zuhlke L., Engel M., Karthikeyan G., Rangarajan S., Mackie P., Cupido B., Mauff K., Islam S., Joachim A., Daniels R. (2015). Characteristics, complications, and gaps in evidence-based interventions in rheumatic heart disease: The Global Rheumatic Heart Disease Registry (the REMEDY study). Eur. Heart J..

[30] Sortsø C., Lauridsen J., Emneus M., Green A., Jensen P.B. (2017). Socioeconomic inequality of diabetes patients’ health care utilization in Denmark. Health Econ. Rev..

[31] Jansen T., Rademakers J., Waverijn G., Verheij R., Osborne R., Heijmans M. (2018). The role of health literacy in explaining the association between educational attainment and the use of out-of-hours primary care services in chronically ill people: A survey study. BMC Health Serv. Res..

[32] Ladwig K., Marten-mittag B., Formanek B., Dammann G. (2000). Gender differences of symptom reporting and medical health care utilization. Eur. J. Epidemiol..

[33] Haskell S.G., Mattocks K., Goulet J., Krebs E., Skanderson M., Leslie D., Justice A.C., Yano E.M., Brandt C. (2011). The Burden of Illness in the First Year Home: Do Male and Female VA Users Differ in Health Conditions and Healthcare Utilization. Women’s Health Issues.

[34] Shalev V., Chodick G., Heymann A.D., Kokia E. (2005). Gender differences in healthcare utilization and medical indicators among patients with diabetes. Public Health.

[35] Vlajinac H., Adanja B., Marinković J., Jarebinski M. (1991). Influence of socio-economic and other factors on rheumatic fever occurrence. Eur. J. Epidemiol..

